# An Overview of the Mechanism of *Penthorum chinense* Pursh on Alcoholic Fatty Liver

**DOI:** 10.1155/2020/4875764

**Published:** 2020-09-16

**Authors:** Xingtao Zhao, Liao Li, Mengting Zhou, Meichen Liu, Ying Deng, Linfeng He, Chaocheng Guo, Yunxia Li

**Affiliations:** ^1^School of Pharmacy, Chengdu University of Traditional Chinese Medicine, Chengdu 611137, China; ^2^Key Laboratory of Standardization for Chinese Herbal Medicine, Ministry of Education, Chengdu 611137, China; ^3^National Key Laboratory Breeding Base of Systematic Research, Development and Utilization of Chinese Medicine Resources, Chengdu 611137, China

## Abstract

Alcohol liver disease (ALD) caused by excessive alcohol consumption is a progressive disease, and alcohol fatty liver disease is the primary stage. Currently, there is no approved drug for its treatment. Abstinence is the best way to heal, but patients' compliance is poor. Unlike other chronic diseases, alcohol fatty liver disease is not caused by nutritional deficiencies; it is caused by the molecular action of ingested alcohol and its metabolites. More and more studies have shown the potential of *Penthorum chinense* Pursh (PCP) in the clinical use of alcohol fatty liver treatment. The purpose of this paper is to reveal from the essence of PCP treatment of alcohol liver mechanism mainly by the ethanol dehydrogenase (ADH) and microsomal ethanol oxidation system-dependent cytochrome P4502E1 (CYP2E1) to exert antilipogenesis, antioxidant, anti-inflammatory, antiapoptotic, and autophagy effects, with special emphasis on its mechanisms related to SIRT1/AMPK, KEAP-1/Nrf2, and TLR4/NF-*κ*B. Overall, data from the literature shows that PCP appears to be a promising hepatoprotective traditional Chinese medicine (TCM).

## 1. Introduction

According to the World Health Organization (WHO), excessive alcohol consumption causes 3 million deaths worldwide each year and is a major cause of death in western and industrialized countries. Alcohol and nonalcohol fatty liver disease have replaced viral hepatitis as the leading cause of disease [1], and among people less than 20 years of age, alcohol is the leading global risk factor for death, with the latest data showing [2] consistently high rates of alcohol-related harm in England. In 2016-2017, one million hospital admissions had a primary or secondary diagnosis related to alcohol consumption [2], causing significant national economic burden. Alcohol abstinence is the best way to fundamentally treat alcohol fatty liver disease [3], but this is poorly complied with patients; regarding ALD medication, it is currently clinically limited to severe alcohol hepatitis with steroids, hexaketocyclohexane (TNF-*α* inhibition), and vitamin E and silymarin (antioxidants), but the clinical effect is not significant and may be related to the complexity of the pathogenesis of alcohol liver disease [4, 5].

Alcohol liver disease (ALD) [1] is a progressive disease that includes a series of dynamic pathologies such as alcohol fatty liver disease (a type of liver damage that is reversible upon abstinence from alcohol), alcohol steatohepatitis, and cirrhosis to liver cancer. Unlike other chronic diseases, alcohol fatty liver disease is not caused by nutritional deficiencies [6], but it is caused by the molecular action of ingested alcohol and its metabolites, etc. After drinking alcohol [7], only about 5–10% passes in nonmetabolic forms through urine (2–4%), exhaled air (2–4%), and skin evaporation (1-2%) and is excreted from the body. The remaining 90–95% is biotransformed in hepatic parenchymal cells (85% of the absorbed dose) and ethanol is excreted in the liver via the ethanol-derived fraction. Dehydrogenase (ADH) (70–80%) and microsomal ethanol oxidation system (MEOS) (20–25%) Cytochrome P4502E1 (CYP2E1) metabolism [8], which induced reduction of NAD^+^ to NADH, inhibits the tricarboxylic acid cycle and *β*-oxidation of fatty acids, in addition to the production of acetaldehyde and lipid peroxidation products which leads to the formation of protein adducts, additionally producing acetaldehyde and lipid peroxidation products form protein adducts that lead to impaired secretion of VLDL-TG. This leads to a barrier to the transport of synthetic lipids, inhibition of fatty acid oxidation, and enhanced lipogenesis, resulting in steatosis, a series of hepatotoxins such as ethanol and the metabolite acetaldehyde further interfering with hepatic parenchymal cells, nonparenchymal cells such as Kupffer cells (KCs), hepatic stellate cells (HSCs), and hepatic sinusoidal endothelial cells (LSECs), as well as the intestinal tract to promote ROS and endotoxin production, which then exacerbate the multiple blows of hepatic lipid accumulation, redox imbalance, inflammatory response, and apoptosis and aggravate the development of the disease [9–11].

According to the therapeutic principle of traditional Chinese medicine (TCM), the treatment of alcohol fatty liver belongs to liver depression and spleen deficiency type, and TCM treatment is multicomponent and multitarget, with low toxicity and side effects, and mostly adopts liver and spleen evacuation, combined with blood activating and stasis resolving drugs, which shows significant therapeutic effect.

In China, *Penthorum chinense* Pursh (PCP) has been used for hundreds of years traditionally with the functions of “promote diuresis and reduce swelling,” “circulation of blood,” “circulation of blood stasis,” and “soothing liver and invigorating spleen,” for alleviating symptoms by excessive intake of alcohol as well as in the treatment of traumatic injury, edema, and liver diseases. Recently, it has a wide range of pharmacological effects, such as antiaging [12], antioxidant [13], anti-inflammatory [14], hypoglycemic [15], antibacterial [12], and promoting immune regulation. Modern studies have shown that PCP protects the liver and treats acute viral hepatitis and chronic liver diseases, such as viral hepatitis, alcoholic hepatitis, and other alcohol liver diseases [13]. 12 Chinese patent pharmaceutical preparations have been approved by the China Food and Drug Administration (CFDA) [16]; PCP and its extract have been developed into tea, drinks or tablets, granules, capsules, and pills in the market under the trade name of “Gansu” in China for treatment of liver diseases [17, 18], which is considered as a potential candidate and can be further developed and used. It is worthy of further study and discussion, so this article provides an overview of its possible active ingredients and related mechanisms, aiming to elucidate its underlying molecular mechanisms.

## 2. The Therapeutic Effects of PCP on Alcohol Fatty Liver Disease

**Figure 1:**
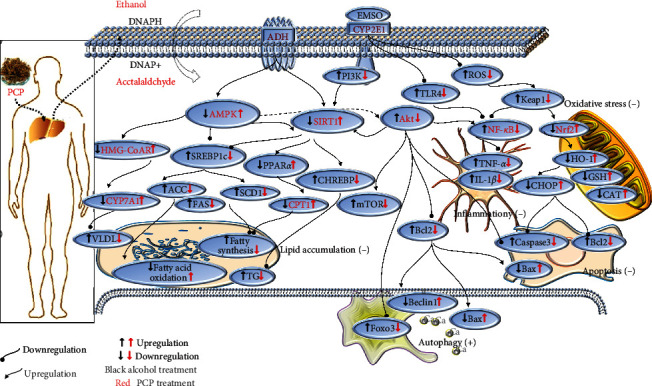
Diagram of the mechanism of PCP on alcohol liver disease.

In recent years, more and more researches have been reported for alcohol liver treatment, which are summarized in Table 1. Expression of activity-regulation-related factors, mainly through SIRT1/AMPK, SREBP-1C, C/EBP, and PPAR-*α*, affects ADH and ALDH activity, further reduces cholesterol synthesis, promotes fatty acid oxidation, and inhibits the oxidation of fatty acids. Increased CYP2E1 activates the KEAP-1/Nrf2-HO-1 signaling pathway to affect relevant oxidative stress factors expression and exert antioxidant effects, which in turn inhibits the MyD88/TLR4/NF-*κ*B signaling pathway and exerts anti-inflammatory effects and finally exerts antiapoptotic effects through PI3K/Akt promotion of B-cell lymphoma (Bcl2), Bcl-2 Associated X Protein (Bax) apoptosis regulator, and inhibition of Caspase 3 protein expression levels, in addition to mediated activation of autophagy which may be related to AMP-activated protein kinase (AMPK) and Sirtuin1 (SIRT1); the specific mechanisms are shown in Figure 1.

### 2.1. Antilipogenesis

The accumulation of fat in the liver is the earliest response to alcohol abuse and is also the first crucial step in the pathogenesis of alcohol liver disease, the early diagnosis of alcohol fatty liver disease is clinically based on the amount of alcohol equivalent to ethanol consumption >40 g/d in men, >20 g/d in women, or >80 g/d in two weeks. Liver fat droplets or total fat cells greater than 5% of liver weight are the criteria for initial diagnosis of steatosis [4]. Importantly, ethanol-induced hepatic lipid accumulation is known to be reversed, and recent studies have shown that PCP had shown great efficacy in the treatment of alcohol fatty liver disease.

Gamma-glutamyl transferase (GGT) is one of the traditional hallmarks of alcoholism [64]. With increased alcohol intake, the blood activity of GGT increases. Aspartate aminotransferase (AST) and alanine aminotransferase (ALT) are involved in the metabolism of amino acids and are ethanol-induced hepatocyte A sensitive indicator of injury. Alkaline phosphatase (ALP) [65] catalyzes the dephosphorylation of different compounds and is a less specific marker of liver injury. Pathological examination of the liver of rats in PCP experimental group detected a significant decrease in the serum content of AST and ALT and a reduction in hepatic fatty infiltration.

The main manifestation of hepatic fat accumulation is the accumulation of triglycerides (TG) [66]. The aqueous extract of PCP played an important role in fat accumulation from chronic alcohol exposure. In adipose tissue, key enzymes were involved in intracellular lipids through upregulation of triacylglycerols, such as adipose triglyceride lipase (ATGL) [67]. Equal amounts of total flavonoids lowered lower-density lipoprotein (LDL), cholesterol (TC), and triglycerides (TG) more significantly than the aqueous extract of chased grass [13], and 95% of the ethanol extracts increased serum high-density lipoprotein (HDL) compared to other volume fractions of ethanol extract group [22]. The fractional ethanol rush extract group was significant, in the dose range of 25–200 g/mL, polyphenols in the 70% ethanol extract. The substances and flavonoids were enriched to a high degree and had significantly stronger hepatoprotective activity than the aqueous extracts [68].

In recent years, there has been great progress in the study of the chemical composition of PCP, the main types of components of polyphenols (flavonoids, coumarins, and lignans), organic acids, sterols, etc. [13]. Among them, polyphenols have shown good efficacy in the treatment of alcohol liver [69].

Quercetin significantly reduced acetyl coenzyme A carboxylase (ACC) activity in rat hepatic suspension cells and decreased fatty acid de novo and TAG synthesis, resulting in VLDL-TAG formation being reduced for lipid-lowering purposes in chronic ethanol-induced alcohol fatty liver disease in rats. Quercetin exerted hepatoprotective effects by lowering LDL, TC, TG, and increased serum HDL [25]; the combination of resveratrol and quercetin reduced peroxidase expression of the proliferator-activated receptor (PPAR) [70] and CCAAT/enhancer-binding protein (C/EBP) [28], via inhibiting the preadipocyte differentiation and exerting potential antiobesity effects, which is primarily associated with upregulation of phosphorylated adenosine monophosphate-activated protein kinase (AMPK) and its substrate ACC levels, and in HepG2 cells [26], quercetin-composed complex increased ATP-binding cassette transporter (ABCA1), cholesterol 7-hydroxylase (CYP7A1), and AMP-activated protein kinase (AMPK) mRNA expression while decreasing sterol regulatory element-binding protein 1c (SREBP-1c) and liver X receptor (LXR) mRNA expression.

Resveratrol was able to inhibit ethanol metabolism and adipogenesis from the head by regulating ethanol dehydrogenase 1B (ADH1B) and acetaldehyde dehydrogenase 2 (ALDH2) mRNA [51, 71] and improving the ratio of NAD^+^/NADH to regulate SIRT1. 3T3-L1 [27] preadipocytes indicated that SIRT1 mediated gene and protein expression of the transcription factors PPAR*γ*, C/EBP, and SREBP-1c, which were involved in the regulation of adipogenesis by resveratrol [71], with PPAR*γ*, PGC-1*α*, and PPAR-*α* being considered as the major adipogenic regulators [72]. In an in vivo animal study, resveratrol treatment increased SIRT1 expression levels and stimulated AMPK activity in the liver of ethanol-fed mice. Resveratrol-mediated increase in SIRT1 and AMPK activity with SREBP-1 inhibition and activation of the peroxisome proliferation-activated receptor coactivator (PGC-1) were associated [73]. Meanwhile, in ethanol-fed mice, resveratrol significantly increased circulating lipofuscin levels and enhanced hepatic lipofuscin receptor mRNA expression (AdipoR1/R2) to prevent the development of alcohol liver steatosis [71].

It has also been shown that the three flavonoids of PCP were induced by free fatty acids (FFA) in a model of hepatic steatosis in HepG2 cells [32]. Pinocembrin-7-O-*β*-d-glucoside (PCBG), Pinocembrin (PCB), and 5-methoxy-pinocembrin-7-O-*β*-d-glucoside (MPG) enhanced phosphorylation of AMPK and silenced mating-type information regulated the expression of SIRT1 and PPAR-*α* and reduced the expression of sterol regulatory SREBP-1c and the downstream targets were fatty acid synthase (FAS). Acetyl coenzyme A carboxylase (ACC) and stearoyl coenzyme A desaturase 1 (SCD1) played a preventive role in hepatic steatosis.

In conclusion, the effect of PCP and its active ingredients on the fat accumulation in alcohol liver was mainly due to the effect of SIRT1/AMPK, SREBP-1C, C/EBP, and PPAR-*α* on the activity of ADH and ALDH to reduce the synthesis of cholesterol and promote the oxidation of fatty acids.

### 2.2. Antioxidant

Excess hepatic acetaldehyde and alcohol oxidation metabolites induce hepatocyte microsomal enzyme cytochrome P450 system activity which promoted high ROS production by a variety of cells including hepatocytes, Kupffer cells, endothelial cells, and infiltrating inflammatory leukocytes, leading to oxidation reduction of imbalance. Antioxidant therapy may be an effective way to correct the imbalance of oxidants and antioxidants in the development of alcohol liver disease and can prevent alcohol liver damage.

Aqueous extracts of PCP [35] had a protective effect against ethanol-induced acute and chronic liver injury and in an ethanol-induced chronic liver injury model. Aqueous extract of PCP downregulated MDA levels in alcohol fatty liver rats by inhibiting CYP2E1-mediated oxidative stress. The potential hepatoprotective effects of SOD were exerted by increasing the activity of SOD, enhancing the scavenging ability of oxygen free radicals and resisting lipid peroxidation. The 95% ethanol extract of PCP increased the liver MDA content and liver SOD content, which had a protective effect on the liver of alcohol rats. The stems of PCP had strong antioxidant activity and low cytotoxicity, making it a better bioactive site. The polyphenols enriched in the ethyl acetate part of PCP directly scavenged ROS and reduced liver enzymes and indirectly increased antioxidant levels. They promoted the production of nuclear factor-like 2 (NRF2), superoxide dismutase-2 (SOD-2), and heme oxygenase-2 (H2O2); HO-1 expression inhibits the Kelch-like ECH associated protein 1 (KEAP-1) expression [36] and thus is resistant to ROS-induced mitochondrial oxidative stress, and quercetin pre-intervention on the hepatocyte alcohol oxidative damage [34] was protective and mediated through Nrf2/HO-1 signaling pathway.

Pinocembrin mitigated ROS accumulation through SIRT3 activation of SOD2 and inhibited KEAP-1 by activating the Nrf2/HO-1 pathway, restored endogenous nonenzymatic (e.g., glutathione (GSH)) depletion, and increased related antioxidant genes (e.g., superoxide dismutase (SOD) and catalase (CAT)) and glutathione peroxidation chemotaxis (GPx) and heme oxygenase (HO-1) activities.

Quercetin inhibited oxidative stress and MDA [39, 40] in HepG2 cells overexpressing CYP2E1 treated with 100 mM ethanol attenuated alcohol-induced increment of hepatic 4-hydroxynonenal (4-HNE) formation with the elevation of hepatic GPX and SOD. Quercetin attenuated CYP2E1-mediated ethanol hepatotoxicity via HO-1 induction, and HO-1-released CO via the activation of the p38 MAPK pathway inhibits ethanol-induced hepatic oxidative damage [47].

The liver is a major site of iron storage and a target organ for iron-induced injury. Hepatic iron content is increased in alcohol fatty liver disease, and hepatic iron can further synergistically amplify ethanol-induced oxidative stress and enhance hepatic tissue and hepatocytes of lipid peroxidative damage, thereby exacerbating fatty liver. In an animal model of alcohol liver, quercetin reduced oxidatively active iron-mediated ROS overproduction in lysosomes and transferrin receptor (TfR) 1 expression and attenuated ethanol-induced hepatic oxidative damage.

Resveratrol significantly reduced alcohol-induced increase in CYP2E1 [41], promoted antioxidant CAT, SOD, GSH, MDA, NADPH quinone oxidoreductase e (NQO1), and glutathione-s-transferase activity (GST), and regulated antioxidation via Nrf2 transcription factor.

In summary, the treatment of alcohol fatty liver disease with PCP exerted antioxidant effects by inhibiting the increase of CYP2E1 and influencing the expression of relevant oxidative stress factors through the KEAP-1/Nrf2-HO-1 signaling pathway.

### 2.3. Anti-Inflammatory

CYP2E1 produces a large number of inflammatory factors through chemotaxis of inflammatory factors, not only ethanol; particularly acetaldehyde in the intestinal tract will destroy tight junction proteins and increase intestinal permeability in vivo and in vitro, via hepatic and intestinal circulation leading to an increase in endotoxin causing induced liver injury.

The water extract of PCP significantly reduced the hepatic lipid accumulation and inflammatory cytokines (e.g., TNF-*α*, IL-6) induced by chronic ethanol exposure. Compared with LPS treatment, the polysaccharide components of PCP protected alcohol fatty liver in rats and exhibited strong anti-inflammatory properties in RAW264.7 cells by inhibiting the release of NO, TNF-*α*, and IL-1*β* activity [14]. The further development of inflammation may lead to liver fibrosis, and total flavonoids of PCP significantly inhibited the decrease of body mass and mitigated further development of liver fibrosis in alcohol rats, then elevated liver coefficient, decreased serum hyaluronic acid (HA), laminin (LN), type III pre-collagen (PCIII) levels, and the content of Hyp in liver tissue, and reduced serum TNF-*α*, IL-6 levels. Moreover, pinocembrin reduced production of the transforming growth factor TGF-*β*, as well as the activation of glycogen synthase kinase 3 via SIRT3/GSK3 to enhance smad protein degradation to treat liver fibrosis [35].

Quercetin inhibited lipopolysaccharide-induced inflammation via the p38MAPK and JNK signaling pathways [45] to inhibit inflammatory mediators (release of PGE2, iNOS, COX-2, TNF-*α*, IL-1*β*, IL-6, and NO) [74]; STAT3 proinflammatory factors play an important role in alcohol liver, and quercetin phosphorylates transcripts by inhibiting signal transducers and activating STAT3, a known PI3K inhibitor, which is also mediated by the TLR4 signaling pathway, thereby disrupting the linkage of the p85 subunit to the adapter protein MyD88 and the TLR4 complex, inhibiting downstream nuclear phosphorylation levels of transcription factor (NF-*κ*B) [48] and protein kinase B (a protein kinase) to treat alcohol-induced liver injury in rats [75]. Quercetin also upregulated the expression of the anti-inflammatory factor IL-10 [43], then inhibiting NLRP3 inflammasome activation and inflammatory factor secretion to maintain liver function in acute alcohol [46].

Resveratrol was able to inhibit TLR4 protein expression [55] by downregulating the mRNA levels of MyD88 and TRAF6, thereby inhibiting TLR4 signaling pathway. It also inhibited the NF-*κ*B/p65 signaling cascade and MAPKs in the liver (p38MAPK, ERK1, ERK2, and ERK5) signaling pathways [54], reducing mRNA levels of proinflammatory mediators and cytokines (IL-1, IL -6, NO, iNOS, and COX-2) [30] to reduce the LPS-induced inflammatory response.

In conclusion, the treatment of alcohol fatty liver disease with PCP mainly exerted anti-inflammatory effects by inhibiting CYP2E1 to attenuate the release of endotoxin and inhibiting MyD88/TLR4/NF-*κ*B signaling pathway.

### 2.4. Autophagy

Autophagy is the phagocytosis of LDs by autophagosomes and their subsequent fusion with lysosomes to form autophagic lysosomes, which is an important pathway for lipid catabolism in lipid droplets, and the promotion of autophagy helps to reduce lipid accumulation in cells.

Four novel autophagy compounds were identified from PCP [76] that activate the AMPK and mTOR-mediated autophagy pathway to restore mitochondrial membrane potential (MMP) and thereby reduce reactive oxygen species (ROS) production in human umbilical cord blood.

Quercetin was able to mediate apoptosis in mature adipocytes by regulating the ERK and JNK pathways [37], and recent studies have shown that quercetin-mediated chronic ethanol-induced mitochondrial autophagy activates AMPK by reducing lipoprotein 2 (PLIN2) levels [60] and extracellular signal-regulated kinase 2 (ERK2), increasing hepatic LC3II, parkin, p62, and the voltage-dependent anion channel 1 (VDAC1) [59], FoxO3a, regulates ethanol-induced autophagy and cell survival. The key factor, quercetin-mediated enhancement of FoxO3a transcriptional activity, was involved in protection against ethanol-induced inhibition of mitochondrial autophagy effects.

Resveratrol increased the levels of microtubule-associated protein LC3-II and Beclin1 [58] and decreased the levels of expression of p62 protein induced autophagy to exert protective effects against alcohol fatty liver disease [57]. Moreover, resveratrol, a well-known SIRT1 agonist, was targeted by FoxO3a genes and increased in primary hepatocytes and mouse liver after treated with ethanol; however, the exact mechanism of action remains to be further studied, which may be related to the increased deacetylation of FoxO3a.

All in all, PCP-mediated activation of autophagy was related to AMPK, SIRT1, and ERK pathways.

### 2.5. Antiapoptosis

Hepatocyte apoptosis induced by ethanol involves oxidative stress and inflammation through induction of associated enzymes (e.g., CYP2E1) and cytokine (e.g., TNF-*α*) production or involvement of death receptors.

The potent hepatoprotective effects of PCP on t-BHP-induced injury in human hepatic L02 cells were mainly mediated by mediating ROS clearance and upregulating the expression of B-cell lymphoma 2 protein (Bcl-2) and Bcl 2-associated X (Bax) [33] and downregulating the caspase cleavage products caspase-7, caspase-9, and PARP to attenuate t-BHP-induced hepatocyte apoptosis [77].

Iron-mediated production of lysosomal ROS leads to lysosomal membrane permeability (LMP) and subsequent release of histone proteases into the cytoplasm, and quercetin induces apoptosis by activating MMP, activating the osteogenic protein/BMP6/SMAD4 pathway, and upregulating iron-regulator expression [37].

Quercetin, a known PI3K inhibitor, prevented alcohol-induced liver injury through the phosphatidylinositol 3-kinase PI3K/Akt/NF-*κ*B and STAT3 signaling pathways and also promoted B-cell lymphoma (Bcl2) and Bcl 2-associated X (Bax) apoptosis regulator, inhibited protein expression levels of caspase 3 and poly ADP ribose polymerase, and decreased apoptosis [46].

Apoptosis is the result of further development of oxidative stress and is mainly executed by caspase 3, and studies have shown that resveratrol attenuated alcohol-induced oxidative stress in rats and then inhibited caspase 3 activity to suppress apoptosis.

In conclusion, the treatment of alcohol liver with PCP mainly inhibited the activity of CYP2E1 enzyme, PI3K/Akt promoted Bcl2, and Bax-related apoptosis regulator, and inhibited the expression of caspase 3 protein to play an antiapoptotic role.

## 3. Discussions

The pathogenesis of alcohol fatty liver is complex, but the essence is mainly through the influence of alcohol dehydrogenase (ADH) (70–80%) and cytochrome P4502E1 (CYP2E1) enzyme activity [78] of microsomal ethanol oxidation system (MEOS) (10–25%), influence of ethanol metabolism; ADH enzyme activity [79] regulates lipid metabolism by affecting SIRT1 [80], AMPK [79], SREBP-1C, ChREBP, and PPAR-*α*. Moreover, CYP2E1 can induce oxidative stress, autophagy, and apoptosis via producing a series of ROS and endotoxin after alcohol [4]; the study shows PCP in the treatment of alcohol fatty liver from the essence of the ADH, CYP2E1 enzyme activity, and then it regulates ethanol metabolism, which is unified with the theory of Chinese medicine “both the symptoms and the treatment.”

Among the relevant regulators that affect lipid metabolism by affecting ADH enzyme activity are SIRT1, AMPK, SREBP-1C, ChREBP, and PPAR-*α*.

SIRT1 [81] is an NAD^+^ dependent histone deacetylase involved in the regulation of many processes, such as mitochondrial biology occurrence, inflammation, intracellular metabolism, resistance, and apoptosis; SIRT1 [82] can be regulated by directly modifying histones or indirectly modulating several transcriptional regulator activity to act as a major metabolic regulator, and increased SIRT1 activity [83] may be caused by blocking ethanol metabolism [NAD^+^]: changes in the [NADH] ratio mediated by Sirt1 activation may involve activation of AMPK [84], and in addition, SIRT1 may enable SREBP-1 [85], the ChREBP deacetylates, thereby inhibiting SREBP-1 [86] and ChREBP activity [87].

AMPK [88] is an intracellular energy sensor and activation of this enzyme inhibits anabolic pathways while promoting lipid decomposition of metabolic processes [89], ADH and/or acute ethanol-induced cardiac contraction, and intracellular Ca^2+^ responses with hyperactivation AMPK signaling cascade [90], phosphorylating downstream targets, including ACC which ultimately promotes long-chain fatty acids of *β* oxidation and leads to decreased accumulation of lipids in tissues, and the increased lipofuscin signaling allows the liver to stimulate AMPK [79] in the liver by lipofuscin resulting in the development of new lipid metabolism [91]. Hepatic fatty acid oxidation is increased [92], thereby preventing hepatic lipid accumulation in ethanol-fed mice after resveratrol, instead of PCP [93].

SREBP-1 [94] is a negative regulator of the ADH gene; sterol regulatory element-binding protein 1 (SREBP-1) and its target transcriptional acetyl coenzyme A carboxylase (ACC), fatty acid synthase (FAS), and stearoyl coenzyme A desaturase levels can be chronically administered by ethanol administration1 (SCD1), and ATP citrate lyase and mitochondrial glyceryl 3-phosphate acyltransferase (GPAT1) can lead to increased fatty acid synthesis and triglyceride accumulation in animal livers.

In addition to SREBP-1c, ChREBP is another factor that regulates sugar and lipid metabolism [95], the ChREBP/SIRT1/ADH axis [96] controls alcohol metabolism, silencing ChREBP can inhibit intrahepatic triglyceride accumulation by activating SIRT1 to reduce ADH acetylation and its enzymatic activity, and in addition, acetaldehyde is a precursor of acetic acid and acetyl coenzyme A (coenzyme A), a source of acetyl groups merged on ChREBP [97], which can effectively alleviate lipid accumulation [98].

The CYP2E1-induced oxidative stress may be related to ethanol-induced inhibition of Akt phosphorylation [99] by reducing its activity affecting the Nrf2/HO-1 signaling pathway, in which NF-*κ*B is an important transcription factor in the development of the inflammatory response, and its activation plays a crucial role in the production of various proinflammatory mediators [100].

Autophagy plays an important role in the pathogenesis of alcohol hepatitis, although it has not been reported in the treatment of alcohol hepatitis with PCP [101].

However, PCP-EE has antiapoptotic, antiaging, and anti-inflammatory properties that protect HaCaT cells from UVB radiation or H_2_O_2_ lethal-induced oxidative stress and increases the promoter activity of the type 1 collagen gene Col1A1 and decreases the UVB radiation or H_2_O_2_ induced oxidative stress induced MMPs, COX-2, IL-6, and hyaluronan [12], suggesting that the treatment of alcohol liver-induced oxidative stress by autophagy is a novel mechanism [102], combined with quercetin, and the therapeutic results of resveratrol in alcohol liver suggest that it may regulate autophagy through the core regulator of autophagy; the transcription factor forkhead box protein O3 (FOXO3a) [103] plays an important role in the pathogenesis of alcohol liver [104], and there is crosstalk between autophagy and apoptosis; PCP and its active ingredients inhibit the PI3K/AKT pathway through SIRT1 activation [105] by promoting Bcl-xl, the expression of Bcl2, and caspases proteins to regulate apoptosis, and it has been shown that Bcl-2 protein not only counteracts the activity of proapoptotic proteins to downregulate apoptosis but also interacts with Beclin-1 to prevent autophagy. The results suggest that there is some crosstalk between autophagy and apoptosis, providing a new thought on the mechanism of action of PCP in the treatment of alcohol liver [106].

Quercetin and resveratrol have been shown to promote autophagy in the treatment of alcoholic liver but recent reports showed that it is reasonable to think that, under mTOR inhibition or caloric restriction, resveratrol could act as an autophagy suppressor instead of behaving as an autophagy inducer. Additionally, because autophagy induced by quercetin or resveratrol is negatively regulated by mTOR, which acts as an autophagy inhibitor or blocker by at least modulating AMPK phosphorylation levels and downregulating HO-1 expression, in this sense, it has been previously shown by other authors that resveratrol induced lysosome leakage. Furthermore, resveratrol-mediated autophagy and apoptosis in cervical cancer cells were related to resveratrol-mediated cathepsin-L release from the lysosomes So, it is feasible to think that, in our experimental model, at least, some part of the proapoptotic signaling could be also mediated by cathepsin release. In this sense, in the quercetin + resveratrol condition, it would be likely that resveratrol is “loading” HepG2 cells with AV (autophagolysosomes) that are permeated by resveratrol. It is harmful to the cytosol (inducing a potent proapoptotic effect). This would also explain why the maximal mTOR inhibition occurs in the quercetin + resveratrol condition; we observed a significant decrease or blockade on the autophagy process [107]; in acute alcoholic fatty liver, the protective effect is played by promoting autophagy to reduce damaged mitochondria and lipid droplets and reduce hepatocyte apoptosis and steatosis. However, in chronic alcoholic fatty liver, autophagy is reduced, leading to lysosome depletion and inhibiting hepatocyte lipid overload, and preventing neutrophil recruitment to the liver; there is a great similarity between them, suggesting that autophagy is an important mechanism of alcoholic liver therapy.

Quercetin, resveratrol, and other flavonoids in PCP have shown good results in the treatment of alcohol liver disease, and the mechanism is similar to that of PCP, and quercetin is also used as an index of quality evaluation for heparin preparations at present, but some studies have proposed that the therapeutic effect of a single ingredient is not as good as that of PCP, which corresponds to the multicomponent, multitarget feature of Chinese medicine, prompting us to look for more quality standards [69].

Recent researches showed that the flavonoids content in the stems of PCP was higher and the antioxidant capacity was stronger [108], which suggested that the pharmacological effects of different medicinal parts were not the same, which can be carried out in the following researches.

With respective clinical applications, quercetin and resveratrol had relatively poor bioavailability due to their low aqueous solubility, short metabolic period, and toxicity. Therefore, extensive efforts have been made to increase the bioavailability and decrease the toxicity of quercetin by encapsulating it in drug delivery systems including silica nanoparticles and so on [109].

## 4. Conclusion

To summarize, this review critically demonstrates that PCP exhibits strong defensive effects against the treatment of alcohol liver, which is mainly concerned with regulating the expression of enzyme activity related ethanol metabolism factors, including SIRT1/AMPK, SREBP-1C, C/EBP, and PPAR-*α* influencing ADH and ALDH activity to reduce cholesterol synthesis and promote the oxidation of fatty acids, the inhibition of CYP2E1 enzyme activity, and the activation of KEAP-1/Nrf2-HO-1 oxidative stress-related signaling pathway to exert antioxidant effects, then inhibiting MyD88/TLR4/NF-*κ*B signaling pathway to exert anti-inflammatory effects and finally promote B-cell lymphoma via PI3K/Akt (Bcl2), Bcl 2-associated X (Bax) apoptosis regulator, and inhibition of caspase 3 protein expression levels to exert antiapoptosis, and additionally mediating activation of autophagy may be related to AMPK and SIRT1. Thus its potential in the mechanism of alcohol fatty liver deserves consideration and further validation.

## Figures and Tables

**Table 1:** Therapeutic effects of PCP and its active ingredients on alcohol fatty liver disease and related mechanisms.

Animal model	Medicine	Dose/treatment	Effect	Mechanism	Reference
Mice fed with high fat diet and alcohol at the dose of 3 g/kg	PCP (95% ethanol)	20, 40, 80 mg/kg 21 d	TG, TC, ALT, AST↓ ↑MDA	Antilipogenesis	[19]
L02 feed with 1.5% FeSO_4_ with 50% ethanol	PCP (50% ethanol)	40 *μ*g/mL 12 d	TG, ALT, AST↓	Antilipogenesis	[20]
Feed with 1.5% FeSO_4_ and 50% ethanol for rats	PCP (95% ethanol) quercetin	4000, 2000 mg/kg 50 mg/kg 6 w	TG, TC, ALT, AST↓ *P. chinense* Pursh played a more significant role than quercetin	Antilipogenesis	[21]
Feed with high fat iron with 52% ethanol, 15% sugar for rats	PCP (35, 75, 95% ethanol)	3 g/kg 4 w	TC, TG, LDL-C, AST↓ 75%, 95% ethanol extract *P. chinense* Pursh played a more significant role	Antilipogenesis	[22]
Rats fed with 1.5% FeSO_4_ and 45% ethanol	PCP (aqueous)	1.67, 8.4, 4.2 g/kg 6 w	TG, CHO, LDL-C, NEAF↓ ALT, AST, TBIL↓ ↑HDL-C	Antilipogenesis	[23]
1.5% ferrous sulfate feed, 50% 8 g/kg alcohol	PCP (aqueous) total flavonoids	2000 mg/kg 800 mg/kg 14 d	TG, TC, ALT, AST↓ total flavonoids played a more significant role than *P. chinense* Pursh	Antilipogenesis	[24]
Fatty acids, cholesterol, neutral lipids, phospholipids, and very-low-density lipoproteins (VLDL) were investigated in rat hepatocyte	Quercetin	50–25 *μ*M	TG, VLDL↓ HMG-CoA-R, ACC↓	Antilipogenesis	[25]
Lipid accumulation in human hepatoma (HepG2 cells)	Quercetin	10 *μ*M 24 h, 48 h	TC, TG, ABCA1↓ HMGCR, SREBP-1c↓ ↑CYP7A1, AMPK*α*2,	Antilipogenesis	[26]
Adipogenesis in 3T3-L1cell	Quercetin	100 Mm 24 h, 48 h	PPAR*γ*, C/EBP*α*↓ ↑AMPK ACC↓ ↑MAPK/ERK1/2 Cytochrome c↓	Antilipogenesis Autophagy	[27]
Adipogenesis and apoptosis in 3T3-L1 cells	Quercetin	10, 20, 40, 80 *μ*M 5, 15, 45, 135 *μ*M 4 w	PPAR*γ*, C/EBP*α*↓ SREBP-1c↓	Antilipogenesis	[28]
Rats ingesting 65% ethanol solution	Resveratrol	250 mg/kg 4 w	TG, ALT, AST↓	Antilipogenesis	[29]
HepG2 cells with 100 *μ*M oleic acid and 87 mM alcohol	Resveratrol	5, 15, 45, 135 *μ*M 24 h	TG↓ ↑AMPK ACC, SREBP-1c, PGC-1*α*↓ ↑PPAR-*α*, Lipin1	Antilipogenesis Antiadipogenic	[30]
Rats ingesting 10% ethanol solution	Quercetin	10 mg/kg 2 w	↑Insulin, adiponectin ↑AMPK ACC↓	Antiadipogenic	[31]
Vitro model of a normal rat's liver cell (BRL-3A).	PCP (ethanol) (70% ethanol)	6.25–100 mg/kg	70% ethanol extract *P. chinense* Pursh played a more significant role than aqueous extract	Antioxidant	[20]
HepG2 cells were induced by free fatty acid (FFA)	Effective flavonone	PCB, MPG (1, 10, 100 *μ*M) PCBG (0.1, 1, 10 *μ*M)	ALT, AST↓ ↑AMPK/SRIT1 ↑PPAR-*α* FAS, ACC, SCD1↓ ↑MDA, GSH-Px	Antioxidant Antilipogenesis	[32]
ABTS, hydroxyl radical scavenging, DPPH tests	PCP (75% ethanol)	0.5–50 mg/kg	DPPH, ABTS, and hydroxyl radicals scavenging↓ lipid peroxidation↓	Antioxidant Antilipogenesis	[12]
*t*-BHP-induced liver damage in LO2 cells	PCP (aqueous)	100, 200, 400 mg/kg 12 h	Caspase-9, caspase-3↓ ↑PARP, Bcl-xl, Bcl-2	Antioxidant Antiapoptotic	[33]
*t*-BHP-induced liver damage in LO2 cells	PCP (aqueous)	50 mg/kg 12 h	↑Nrf2, HO-1, SOD-2 KEAP-1↓ ↑Bcl-2 E50M60 subfraction was more significant than *P. chinense* at 200 *μ*g/mL	Antioxidant Antiapoptotic	[34]
Ethanol gavage (4.7 g/kg) every 12 h for a total of three doses	PCP (aqueous)	5.2, 10.3 g/kg 7 d	TG, ATGL, ALT, AST↓ CYP2E1↓ ↑MDA, GSH, SOD, CAT TNF-*α*, IL-6↓	Antilipogenesis Antioxidant Anti-inflammatory	[35]
Mice were fed a Lieber-De Carli liquid diet containing alcohol	PCP (aqueous)	5, 10, 30 g/kg 4 w	ALT, AST↓ TNF-*α*, IL-6↓ ↑MDA, GSH, SOD, GPx CYP2E1↓ ↑Nrf2/HO-1	Antioxidant Anti-inflammatory	[36]
RAW264.7 cell with LPS (1 ng/mL)	PCP (80% ethanol) PCPP, PCPP1a	15, 30, 60, 120 *μ*g/mL 24 h	NO, TNF-*α*, IL-1*β*↓	Anti-inflammatory	[14]
Human LX-2 cells and rat HSC-T6 cells	PCP (aqueous)	25, 50, 100 mg/kg 24 h	Collagen I, *α*-SMA, TGF-*β*1↓ ↑SIRT3, SOD2, GSK3*β* PI3K-Akt↓	Antioxidant	[35]
Male C57BL/6J mice fed Lieber-De Carli diets containing ethanol (30% of total calories) hepatocytes with ethanol (100 mM)	Quercetin	100 *μ*M 24 h	TC, TG, ALT, AST↓ ↑GSH, MDA, TfR1 ↑BMP6/SMAD4, ↑hepcidin	Antioxidant Autophagy	[37]
Chronic alcohol (30% of total calories) or iron (0.2%)-fed adult male C57BL/J mice	Quercetin	100 *μ*M	↑Metal transporter 1, zinc transporter member 14, mucolipin 1, transferrin receptor 1	Hepatoprotective Antioxidant	[38]
Mouse primary hepatocytes were incubated with ethanol (100 mM)	Quercetin	100 *μ*M	ALT↓ ↑HDL ↑ROS, SOD, MDA, GSH	Hepatoprotective Antioxidant	[39]
Male C57BL/6J mice fed Lieber-De Carli diets containing ethanol	Quercetin	100 *μ*M	AST, ALT, LDH↓ ↑MDA, GSH, GSSH caspase-3↓	Antiapoptosis	[40]
MS rats with 30% sucrose in drinking water	Quercetin	0.95 mg/kg	TG, HOMA↓ ↑GSH, Nrf2	Antioxidant	[41]
Ethanol (4.0 g/kg) to rats	Quercetin	100 mg/kg 9 d	AST, LDH↓ CYP2E1↓ ↑MDA, GST, GPx, CAT, ↑HO-1	Antioxidant	[42]
Ethanol-incubated primary rat hepatocytes (100 mM)	Quercetin	50 *μ*M 2 h	AST, LDH↓ ↑MDA, GSH, SOD, CAT ↑Nrf-2/HO-1	Antioxidant	[43]
HepG2 cell model induced by ethanol in vitro	Quercetin	25 *μ*M	CYP2E1↓ ↑MDA, 4-HNE, GPx ↑Nrf2/HO-1	Antioxidant	[44]
RAW 264.7 cells with LPS (100 ng/ml)	Quercetin	20 *μ*M 24 h	TNF-*α*, IL-1*β*, IL-6, MyD88, PGE2, iNOS, COX-2↓ ↑HO–1 PI3K/Akt/NF-*κ*B, TRAF6 JNK, p38MAPK↓	Anti-inflammatory Antioxidant	[45]
Mice fed with 35%, 40%, 52% v/v ethanol (3 g/kg)	Quercetin	60 mg/kg 3 w	ALT, AST, TG↓ ↑TBIL TNF-*α*, IL-1*β*, IL-2, IL-6↓ ↑IL-10 ↑SOD, GSH-Px, MDA ↑Bcl-xl, Bcl-2 Caspase-3↓ PI3K/Akt/NF-*κ*B↓ ↑STAT3, PARP	Antioxidant Anti-inflammatory Antilipophagy Antiapoptosis	[46]
Ethanol-dosed adult male Balb/c mice (5.0 g/kg) or primary rat hepatocytes (100 mM)	Quercetin	2.5 mg/kg	AST, ALT, LDH↓ ↑GSH, SOD, MDA TNF-*α*, IL-6↓ p38 MAPK↓ ↑HO-1	Antioxidant Anti-inflammatory	[43]
Treated with 5% alcohol for 24 h HepG2 cells	Quercetin	10, 20 *μ*M 1 h	ALT, AST↓ ↑ROS, MDA, GSH NO, TNF-*α*↓ ↑Nrf2/HO-1	Antioxidant Anti-inflammatory	[44]
25 patients with AH/rats with 50% (v/v) ethanol 5 g/kg body weight every 12 h three times	Quercetin	100 mg/kg 14 d	ALT, AST↓ ↑MDA, GSH-Px TNF-*α*, IL-1*β*, IL-18, NLRP3↓ ↑IL-10 ↑Nrf2/HO-1 ASC, caspase-1↓	Antioxidant Anti-inflammatory	[47]
Pregnant rats received 1 ml/day of 40% v/v ethanol (4 g/kg)	Quercetin	50 mg/kg 21 d	↑MDA, PC, GSH, SOD, CAT TNF-*α*, IL-1*β*, IL-6, NF-*κ*B↓	Antioxidant Anti-inflammatory	[48]
BALB/c mice had induced liver fibrosis by carbon tetrachloride (CCl4)	Quercetin	50 mg/kg 8 w	TNF-*α*, IL-1*β*, IL-6, MCP-1↓	Anti-inflammatory	[45]
Peripheral blood of 15 healthy male and female nonsmoking and nondrinking donors	Resveratrol	5, 25, 50 *μ*M	↑ADH1B, ALDH2	Antioxidant	[49]
Mice fed with 3 gavages of calories of dextrin-maltose (MP biomedicals) or ethanol at 3.5 g/kg	Resveratrol	50 *μ*M	AST, ALT, TG↓ ↑ADH, NADH/NAD^+^ ↑SIRT1, AMPK, ChREBP, FAS, SCD1↓	Antioxidant	[50]
Rats were fed either control or ethanol liquid diets containing 0.8, 1.6, and 2.4 g/kg ethanol	Resveratrol	100 mg/kg 22 w	↑ALDH2, ADH, SIRT1 CYP2E1↓	Antioxidant	[51]
Ethanol-induced oxidative DNA damage in human peripheral lymphocytes	Resveratrol	5, 25, 50 *μ*M	↑ADH1B, ALDH2	Antioxidant	[52]
*t*-BHP-induced liver damage in LO2 cells	Resveratrol	25, 50, 75 *μ*M	↑SOD, GPx, GR, NQO1, GST, ↑Nrf2	Antioxidant	[53]
Mice administered alcohol in drinking 10% up to 40% v/v	Resveratrol	10 mg/ml 4 w	TG↓ TNF-*α*, IL-1↓	Anti-inflammatory	[30]
Lipopolysaccharides-induced rats' model	Resveratrol	3.125, 6.25, 12, 5 *μ*M 25 h	ALT, AST, ALP, CHO↓ NF-*κ*B/p65, TLR4, MyD88, TRAF6↓ TNF-*α*, IL-1, IL-1*β*, IL-6↓ NO, iNOS, COX-2↓ ↑MAPKs ↑IL-10 PI3K/Akt↓	Anti-inflammatory	[54]
Mice with carbon tetrachloride (CCl4) induced liver fibrosis	Resveratrol	10, 20, 30 mg/kg	ALT, AST↓ NLRP3, IL-1*β*, IL-18↓ ↑Caspase1	Anti-inflammatory	[55]
Mice with carbon tetrachloride (CCl4) induced liver fibrosis	Resveratrol	50 mg/kg	ALT, AST↓ TNF-*α*, Akt/NF-*κ*B (I*κ*B)↓	Anti-inflammatory	[56]
Male Foxo3a/mice administered 33% (v/v) ethanol at a total dose of 4.5 g/kg	Resveratrol	6, 12 mg/kg 16 h	↑Ulk1, Atg5, 7, 14 ↑Vps34, LC3II, beclin 1 ↑FoxO3a	Autophagy	[57]
AFL mice fed with an ethanol Lieber-De Carli liquid diet, and HepG2 cells in the presence of oleic acid and alcohol	Resveratrol	10, 30, 100 mg/kg	ALT, AST, TG, LDL-C↓ ↑HDL-C ↑LC3-II, P62	Autophagy	[58]
Mice were fed 30% Lieber-De Carli liquid diet containing alcohol	Quercetin	100 mg/kg 15 w	↑LC3II, parkin, p62 ↑VDAC1, FoxO3a ↑AMPK, ERK2	Autophagy	[59]
Pair fed with liquid diets containing ethanol (28% of total calories) and treated mice	Quercetin	100 mg/kg 12 w	ALT, AST, TC, TG↓ ↑AMPK, ERK, PLIN2 ↑LC3II, p62	Autophagy	[60]
*t*-BHP-induced liver damage in LO2 cells	Quercetin	50 *μ*M 12 h	↑Nrf2, HO-1, SOD-2 KEAP-1↓ ↑BcL-2	Autophagy	[61]
Mice administered alcohol in drinking 6% up to 20% v/v	Resveratrol	250 mg/kg 16 w	ALT, AST↓ ↑MDA, SOD, CAT, GPX CYP2E1↓ caspase 3↓	Antioxidant Antiapoptotic	[62]
Human hepatocyte Chang cell line induced by ethanol	Resveratrol	10 *μ*M 6 h	↑SIRT1 Caspase-12↓ ↑ADH-2, ALDH-2, ↑GRP78, p-IRE1*α*, p-eIF2*α* p-PERK, ATF4↓ caspase-3/12↓ ↑CHOP, Bcl-xl	Antiapoptotic	[51, 63]

Note: TG, triglyceride; TC, serum total cholesterol; TBil, total bilirubin; LDL-C, low-density lipoprotein cholesterol; HDL-C, high-density lipoprotein cholesterol; VLDL, very low-density lipoprotein cholesterol; VDACT1, voltage-dependent anion channel; DBIL, direct bilirubin; HMGCR, 3-hydroxy-3-methylglutaryl-coenzyme A reductase; ALT, alanine aminotransferase; AST, aspartate transaminase; ADH, alcohol dehydrogenase; ALDH, aldehyde dehydrogenase; CYP2E1, cytochrome P450 2E1; ABCA1, ATP-binding cassette transporter; CYP7A1, cholesterol 7-hydroxylase; MDA, malondialdehyde; FAS, fatty acid synthase; ACC, acetyl coenzyme A carboxylase; SCD1, stearoyl coenzyme A desaturase 1; GSH, glutathione; SOD, superoxide dismutase; CAT, catalase; GPx, glutathione peroxidation chemotaxis; HO-1, heme oxygenase; NQO1, NADPH quinone oxidoreductase 1; COX-2, cyclooxygenase-2; IL-1, interleukin-1; IL-6, interleukin-6; IL-10, interleukin-10; TNF-*α*, tumor necrosis factor alpha; TLR4, toll-like receptor 4; TRAF6, tumor necrosis factor receptor-associated factor; AMPK, AMP-activated protein kinase; SIRT1, sirtuin1; ERK, extracellular signal-regulated kinase; PPAR-*α*, proliferator-activated receptor alpha; C/EBP, CCAAT/enhancer-binding protein; HA, hyaluronic acid; LN, laminin; PC III, procollagen III; PCIII, type III pre-collagen; LC3-II, light chain 3-II; Bcl2, B-cell lymphoma.

## Data Availability

All the references can be found on PubMed, the citations are marked in the articles, and the corresponding references are listed in the references list.

## References

[1] Browning J. D., Horton J. D. (2004). Molecular mediators of hepatic steatosis and liver injury. *Journal of Clinical Investigation*.

[2] Williams R., Alexander G., Aspinall R. (2018). Gathering momentum for the way ahead: fifth report of the lancet standing commission on liver disease in the UK. *The Lancet*.

[3] Singal A. K., Bataller R., Ahn J., Kamath P. S., Shah V. H. (2018). ACG clinical guideline: alcoholic liver disease. *American Journal of Gastroenterology*.

[4] Stickel F., Datz C., Hampe J., Bataller R. (2017). Pathophysiology and management of alcoholic liver disease: update 2016. *Gut and Liver*.

[5] Gala K. S., Vatsalya V. (2020). Emerging noninvasive biomarkers, and medical management strategies for alcoholic hepatitis: present understanding and scope. *Cells*.

[6] Ishak K. G., Zimmerman H. J., Ray M. B. (1991). Alcoholic liver disease: pathologic, pathogenetic and clinical aspects. *Alcoholism: Clinical and Experimental Research*.

[7] Rocco A., Compare D., Angrisani D. (2014). Alcoholic disease: liver and beyond. *World Journal of Gastroenterology*.

[8] Teschke R. (2018). Alcoholic liver disease: alcohol metabolism, cascade of molecular mechanisms, cellular targets, and clinical aspects. *Biomedicines*.

[9] Seth D., Haber P. S., Syn W.-K., Diehl A. M., Day C. P. (2011). Pathogenesis of alcohol-induced liver disease: classical concepts and recent advances. *Journal of Gastroenterology and Hepatology*.

[10] Kong L. Z., Chandimali N., Han Y. H. (2019). Pathogenesis, early diagnosis, and therapeutic management of alcoholic liver disease. *International Journal of Molecular Sciences*.

[11] García-Ruiz C., Kaplowitz N., Fernandez-Checa J. C. (2013). Role of mitochondria in alcoholic liver disease. *Current Pathobiology Reports*.

[12] Jeong D., Lee J., Park S. H. (2019). Antiphotoaging and antimelanogenic effects of *Penthorum chinense* Pursh ethanol extract due to antioxidant and autophagy-inducing properties. *Oxidative Medicine and Cellular Longevity*.

[13] Wang A., Li M., Huang H. (2020). A review of *Penthorum chinense* Pursh for hepatoprotection: traditional use, phytochemistry, pharmacology, toxicology and clinical trials. *Journal of Ethnopharmacology*.

[14] Lin L. M., Zhao L. J., Deng J. (2018). Enzymatic extraction, purification, and characterization of polysaccharides from *Penthorum chinense* Pursh: natural antioxidant and anti-inflammatory. *BioMed Research International*.

[15] Huang D., Jiang Y., Chen W., Yao F., Huang G., Sun L. (2015). Evaluation of hypoglycemic effects of polyphenols and extracts from *Penthorum chinense*. *Journal of Ethnopharmacology*.

[16] Wang A., Lin L., Wang Y. (2015). Traditional Chinese herbal medicine *Penthorum chinense* Pursh: a phytochemical and pharmacological review. *The American Journal of Chinese Medicine*.

[17] Qu Y., Zong L., Shen L. (2011). Effects of Gansu on oxidative stress and expressions of collagens in rats with CCl4-induced liver fibrosis. *Chinese Journal of Gastroenterology*.

[18] Yang Z., Wang J.-h., Zhao T.-j. (2018). Meta analysis of the curative effect of Gansu Granule on chronic hepatitis B. *Chinese Journal of Modern Medicine*.

[19] Hu X.-y., Wei M., Yuan Y.-F., Yang W.-x. (2015). Experimental study on alcoholic fatty liver of mice treated with the southwest military medical. *Chinese Medicine*.

[20] Zhang T.-T., Xu X.-L., Jiang M.-H., Jiang J.-G. (2013). Hepatoprotective function of *Penthorum chinense* Pursh. *Food & Function*.

[21] Yong T., Zhang C., li G.-c., Chonglin Y. (2016). Effect of ethanol extract of *Herba edulis* on alcoholic fatty liver in rats. *Chinese Traditional Patent Medicine*.

[22] Lu Q., Jiang M.-H., Jiang J.-G., Zhang R.-F., Zhang M.-W. (2012). Isolation and identification of compounds from *Penthorum chinense* Pursh with antioxidant and antihepatocarcinoma properties. *Journal of Agricultural and Food Chemistry*.

[23] Xiao L.-p. (2015). *Master*.

[24] Yuan Y., Ou X. (2018). Research on acute toxicity of *Penthorum chinense* Pursh. Total flavonoids and therapeutic effect on AFL rats. *Medicinal Plant*.

[25] Gnoni G. V., Paglialonga G., Siculella L. (2009). Quercetin inhibits fatty acid and triacylglycerol synthesis in rat-liver cells. *European Journal of Clinical Investigation*.

[26] Leng E., Xiao Y., Mo Z. (2018). Synergistic effect of phytochemicals on cholesterol metabolism and lipid accumulation in HepG2 cells. *BMC Complementary and Alternative Medicine*.

[27] Ahn J., Lee H., Kim S., Park J., Ha T. (2008). The anti-obesity effect of quercetin is mediated by the AMPK and MAPK signaling pathways. *Biochemical and Biophysical Research Communications*.

[28] Yang J.-Y., Della-Fera M. A., Rayalam S. (2008). Enhanced inhibition of adipogenesis and induction of apoptosis in 3T3-L1 adipocytes with combinations of resveratrol and quercetin. *Life Sciences*.

[29] Ma Z., Zhang Y., Li Q. (2017). Resveratrol improves alcoholic fatty liver disease by downregulating HIF-1alpha expression and mitochondrial ROS production. *PLoS One*.

[30] Tang L. Y., Chen Y., Rui B. B., Hu C. M. (2016). Resveratrol ameliorates lipid accumulation in HepG2 cells, associated with down-regulation of lipin1 expression. *Canadian Journal of Physiology and Pharmacology*.

[31] Seo M.-J., Lee Y.-J., Hwang J.-H., Kim K.-J., Lee B.-Y. (2015). The inhibitory effects of quercetin on obesity and obesity-induced inflammation by regulation of MAPK signaling. *The Journal of Nutritional Biochemistry*.

[32] Guo W. W., Wang X., Chen X. Q. (2018). Flavonones from *Penthorum chinense* ameliorate hepatic steatosis by activating the SIRT1/AMPK pathway in HepG2 cells. *International Journal of Molecular Sciences*.

[33] Wang M., Zhang X. J., Feng R. (2017). Hepatoprotective properties of *Penthorum chinense* Pursh against carbon tetrachloride-induced acute liver injury in mice. *Chinese Medicine*.

[34] Wang A., Wang S., Jiang Y., Chen M., Wang Y., Lin L. (2016). Bio-assay guided identification of hepatoprotective polyphenols from *Penthorum chinense* Pursh on *t*-BHP induced oxidative stress injured L02 cells. *Food & Function*.

[35] Zhou F., Wang A., Li D., Wang Y., Lin L. (2018). Pinocembrin from *Penthorum chinense* Pursh suppresses hepatic stellate cells activation through a unified SIRT3-TGF-*β*-Smad signaling pathway. *Toxicology and Applied Pharmacology*.

[36] Cao Y.-W., Jiang Y., Zhang D.-Y. (2015). Protective effects of *Penthorum chinense* Pursh against chronic ethanol-induced liver injury in mice. *Journal of Ethnopharmacology*.

[37] Tang Y., Li Y., Yu H. (2014). Quercetin prevents ethanol-induced iron overload by regulating hepcidin through the BMP6/SMAD4 signaling pathway. *The Journal of Nutritional Biochemistry*.

[38] Tang Y., Li Y., Yu H. (2014). Quercetin attenuates chronic ethanol hepatotoxicity: implication of “free” iron uptake and release. *Food and Chemical Toxicology*.

[39] Li Y., Deng Y., Tang Y. (2014). Quercetin protects rat hepatocytes from oxidative damage induced by ethanol and iron by maintaining intercellular liable iron pool. *Human & Experimental Toxicology*.

[40] Li Y., Chen M., Xu Y. (2016). Iron-mediated lysosomal membrane permeabilization in ethanol-induced hepatic oxidative damage and apoptosis: protective effects of quercetin. *Oxidative Medicine and Cellular Longevity*.

[41] Rubio-Ruiz M. E., Guarner-Lans V., Cano-Martinez A. (2019). Resveratrol and quercetin administration improves antioxidant DEFENSES and reduces fatty liver in metabolic syndrome rats. *Molecules*.

[42] Tang Y., Tian H., Shi Y. (2013). Quercetin suppressed CYP2E1-dependent ethanol hepatotoxicity via depleting heme pool and releasing CO. *Phytomedicine*.

[43] Li Y., Gao C., Shi Y. (2013). Carbon monoxide alleviates ethanol-induced oxidative damage and inflammatory stress through activating p38 MAPK pathway. *Toxicology and Applied Pharmacology*.

[44] Oliva J., Bardag-Gorce F., Tillman B., French S. W. (2011). Protective effect of quercetin, EGCG, catechin and betaine against oxidative stress induced by ethanol in vitro. *Experimental and Molecular Pathology*.

[45] Li X., Jin Q., Yao Q. (2018). The flavonoid quercetin ameliorates liver inflammation and fibrosis by regulating hepatic macrophages activation and polarization in mice. *Frontiers in Pharmacology*.

[46] Zhu M., Zhou X., Zhao J. (2017). Quercetin prevents alcohol-induced liver injury through targeting of PI3K/Akt/nuclear factor-*κ*B and STAT3 signaling pathway. *Experimental and Therapeutic Medicine*.

[47] Liu S., Tian L., Chai G., Wen B., Wang B. (2018). Targeting heme oxygenase-1 by quercetin ameliorates alcohol-induced acute liver injury via inhibiting NLRP3 inflammasome activation. *Food & Function*.

[48] Ince E. (2020). The protective effect of quercetin in the alcohol-induced liver and lymphoid tissue injuries in newborns. *Molecular Biology Reports*.

[49] Yan Y., Yang J.-Y., Mou Y.-H., Wang L.-H., Zhou Y.-N., Wu C.-F. (2012). Differences in the activities of resveratrol and ascorbic acid in protection of ethanol-induced oxidative DNA damage in human peripheral lymphocytes. *Food and Chemical Toxicology*.

[50] Luo G., Huang B., Qiu X. (2017). Resveratrol attenuates excessive ethanol exposure induced insulin resistance in rats via improving NAD^+^/NADH ratio. *Molecular Nutrition & Food Research*.

[51] Liu L., Fan Z., Tang Y., Ke Z. (2014). The resveratrol attenuates ethanol-induced hepatocyte apoptosis via inhibiting ER-related caspase-12 activation and PDE activity in vitro. *Alcoholism: Clinical and Experimental Research*.

[52] Xue L., Zhu W., Yang F. (2019). Appropriate dose of ethanol exerts anti-senescence and anti-atherosclerosis protective effects by activating ALDH2. *Biochemical and Biophysical Research Communications*.

[53] Rubiolo J. A., Mithieux G., Vega F. V. (2008). Resveratrol protects primary rat hepatocytes against oxidative stress damage: activation of the Nrf2 transcription factor and augmented activities of antioxidant enzymes. *European Journal of Pharmacology*.

[54] Wang G., Hu Z., Fu Q. (2017). Resveratrol mitigates lipopolysaccharide-mediated acute inflammation in rats by inhibiting the TLR4/NF-*κ*Bp65/MAPKs signaling cascade. *Scientific Reports*.

[55] Li F., Yang Y., Yang L. (2017). Resveratrol alleviates FFA and CCl4 induced apoptosis in HepG2 cells via restoring endoplasmic reticulum stress. *Oncotarget*.

[56] Zhang D.-Q., Sun P., Jin Q. (2016). Resveratrol regulates activated hepatic stellate cells by modulating NF-*κ*B and the PI3K/Akt signaling pathway. *Journal of Food Science*.

[57] Ni H.-M., Du K., You M., Ding W.-X. (2013). Critical role of FoxO3a in alcohol-induced autophagy and hepatotoxicity. *The American Journal of Pathology*.

[58] Tang L., Yang F., Fang Z., Hu C. (2016). Resveratrol ameliorates alcoholic fatty liver by inducing autophagy. *The American Journal of Chinese Medicine*.

[59] Yu X., Xu Y., Zhang S. (2016). Quercetin attenuates chronic ethanol-induced hepatic mitochondrial damage through enhanced mitophagy. *Nutrients*.

[60] Zeng H., Guo X., Zhou F. (2019). Quercetin alleviates ethanol-induced liver steatosis associated with improvement of lipophagy. *Food and Chemical Toxicology*.

[61] Weng C.-J., Chen M.-J., Yeh C.-T., Yen G.-C. (2011). Hepatoprotection of quercetin against oxidative stress by induction of metallothionein expression through activating MAPK and PI3K pathways and enhancing Nrf2 DNA-binding activity. *New Biotechnology*.

[62] Peiyuan H., Zhiping H., Chengjun S. (2017). Resveratrol ameliorates experimental alcoholic liver disease by modulating oxidative stress. *Evidence-Based Complementary and Alternative Medicine*.

[63] Bujanda L., Garcia-Barcina M., Gutierrez-de Juan V. (2006). Effect of resveratrol on alcohol-induced mortality and liver lesions in mice. *BMC Gastroenterology*.

[64] Niemelä O. (2016). Biomarker-based approaches for assessing alcohol use disorders. *International Journal of Environmental Research and Public Health*.

[65] Wang M., Ma L.-J., Yang Y., Xiao Z., Wan J.-B. (2019). *n*-3 polyunsaturated fatty acids for the management of alcoholic liver disease: a critical review. *Critical Reviews in Food Science and Nutrition*.

[66] Venkatesan S., Ward R. J., Peters T. J. (1988). Effect of chronic ethanol feeding on the hepatic secretion of very-low-density lipoproteins. *Biochimica et Biophysica Acta (BBA)–Lipids and Lipid Metabolism*.

[67] Hu X., Wei M., Yuan Y., Yang W. (2015). Experimental study of *Penthorum chinense* Pursh on mouse with alcoholic fatty liver disease. *Medicinal Plant*.

[68] He L., Zhang S., Luo C. (2019). Functional teas from the stems of *Penthorum chinense* Pursh.: phenolic constituents, antioxidant and hepatoprotective activity. *Plant Foods for Human Nutrition*.

[69] Guo W., Jiang Y., Chen X. (2015). Identification and quantitation of major phenolic compounds from *Penthorum chinense* Pursh. by HPLC with tandem mass spectrometry and HPLC with diode array detection. *Journal of Separation Science*.

[70] Ye G., Gao H., Wang Z. (2019). PPAR*α* and PPAR*γ* activation attenuates total free fatty acid and triglyceride accumulation in macrophages via the inhibition of Fatp1 expression. *Cell Death & Disease*.

[71] Ajmo J. M., Liang X., Rogers C. Q., Pennock B., You M. (2008). Resveratrol alleviates alcoholic fatty liver in mice. *American Journal of Physiology-Gastrointestinal and Liver Physiology*.

[72] Ye G., Chen G., Gao H. (2019). Resveratrol inhibits lipid accumulation in the intestine of atherosclerotic mice and macrophages. *Journal of Cellular and Molecular Medicine*.

[73] Yang X., Xu S., Qian Y., Xiao Q. (2017). Resveratrol regulates microglia M1/M2 polarization via PGC-1*α* in conditions of neuroinflammatory injury. *Brain, Behavior, and Immunity*.

[74] Houghton M. J., Kerimi A., Tumova S., Boyle J. P., Williamson G. (2018). Quercetin preserves redox status and stimulates mitochondrial function in metabolically-stressed HepG2 cells. *Free Radical Biology and Medicine*.

[75] Endale M., Park S.-C., Kim S. (2013). Quercetin disrupts tyrosine-phosphorylated phosphatidylinositol 3-kinase and myeloid differentiation factor-88 association, and inhibits MAPK/AP-1 and IKK/NF-*κ*B-induced inflammatory mediators production in RAW 264.7 cells. *Immunobiology*.

[76] Sun X., Wu A., Kwan Law B. Y. (2020). The active components derived from *Penthorum chinense* Pursh protect against oxidative-stress-induced vascular injury via autophagy induction. *Free Radical Biology and Medicine*.

[77] Hu Y., Wang S., Wang A., Lin L., Chen M., Wang Y. (2015). Antioxidant and hepatoprotective effect of *Penthorum chinense* Pursh extract against t-BHP-induced liver damage in L02 cells. *Molecules*.

[78] Song B.-J., Abdelmegeed M. A., Cho Y.-E. (2019). Contributing roles of CYP2E1 and other cytochrome P450 isoforms in alcohol-related tissue injury and carcinogenesis. *Advances in Experimental Medicine and Biology*.

[79] Srinivasan M. P., Bhopale K. K., Amer S. M. (2019). Linking dysregulated AMPK signaling and ER stress in ethanol-induced liver injury in hepatic alcohol dehydrogenase deficient deer mice. *Biomolecules*.

[80] Yaku K., Okabe K., Gulshan M., Takatsu K., Okamoto H., Nakagawa T. (2019). Metabolism and biochemical properties of nicotinamide adenine dinucleotide (NAD) analogs, nicotinamide guanine dinucleotide (NGD) and nicotinamide hypoxanthine dinucleotide (NHD). *Scientific Reports*.

[81] Ding R.-B., Bao J., Deng C.-X. (2017). Emerging roles of SIRT1 in fatty liver diseases. *International Journal of Biological Sciences*.

[82] Jung T. W., Lee K.-T., Lee M. W., Ka K.-H. (2012). SIRT1 attenuates palmitate-induced endoplasmic reticulum stress and insulin resistance in HepG2 cells via induction of oxygen-regulated protein 150. *Biochemical and Biophysical Research Communications*.

[83] You M., Cao Q., Liang X., Ajmo J. M., Ness G. C. (2008). Mammalian sirtuin 1 is involved in the protective action of dietary saturated fat against alcoholic fatty liver in mice. *The Journal of Nutrition*.

[84] Chang C., Su H., Zhang D. (2015). AMPK-dependent phosphorylation of GAPDH triggers Sirt1 activation and is necessary for autophagy upon glucose starvation. *Molecular Cell*.

[85] Zhang N., Hu Y., Ding C. (2017). Salvianolic acid B protects against chronic alcoholic liver injury via SIRT1-mediated inhibition of CRP and ChREBP in rats. *Toxicology Letters*.

[86] Defour A., Dessalle K., Castro Perez A. (2012). Sirtuin 1 regulates SREBP-1c expression in a LXR-dependent manner in skeletal muscle. *PLoS One*.

[87] You M., Liang X., Ajmo J. M., Ness G. C. (2008). Involvement of mammalian sirtuin 1 in the action of ethanol in the liver. *American Journal of Physiology-Gastrointestinal and Liver Physiology*.

[88] Hardie D. G. (2003). Minireview: the AMP-activated protein kinase cascade: the key sensor of cellular energy status. *Endocrinology*.

[89] Carling D. (2004). The AMP-activated protein kinase cascade—a unifying system for energy control. *Trends in Biochemical Sciences*.

[90] Hintz K. K., Relling D. P., Saari J. T. (2003). Cardiac overexpression of alcohol dehydrogenase exacerbates cardiac contractile dysfunction, lipid peroxidation, and protein damage after chronic ethanol ingestion. *Alcoholism: Clinical & Experimental Research*.

[91] Hu M., Wang F., Li X. (2012). Regulation of hepatic lipin-1 by ethanol: role of AMP-activated protein kinase/sterol regulatory element-binding protein 1 signaling in mice. *Hepatology*.

[92] Guo R., Ren J., Ren J. (2010). Alcohol dehydrogenase accentuates ethanol-induced myocardial dysfunction and mitochondrial damage in mice: role of mitochondrial death pathway. *PLoS One*.

[93] Jiang Z., Zhou J., Zhou D., Zhu Z., Sun L., Nanji A. A. (2015). The adiponectin-SIRT1-AMPK pathway in alcoholic fatty liver disease in the rat. *Alcoholism: Clinical and Experimental Research*.

[94] He L., Simmen F. A., Ronis M. J. J., Badger T. M. (2004). Post-transcriptional regulation of sterol regulatory element-binding protein-1 by ethanol induces class I alcohol dehydrogenase in rat liver. *Journal of Biological Chemistry*.

[95] Sato S., Jung H., Nakagawa T. (2016). Metabolite regulation of nuclear localization of carbohydrate-response element-binding protein (ChREBP): role of amp as an allosteric inhibitor. *Journal of Biological Chemistry*.

[96] Liangpunsakul S. (2015). Carbohydrate-responsive element-binding protein, sirtuin 1, and ethanol metabolism: a complicated network in alcohol-induced hepatic steatosis. *Hepatology*.

[97] Gao L., Shan W., Zeng W. (2016). Carnosic acid alleviates chronic alcoholic liver injury by regulating the SIRT1/ChREBP and SIRT1/p66shc pathways in rats. *Molecular Nutrition & Food Research*.

[98] Marmier S., Dentin R., Daujat-Chavanieu M. (2015). Novel role for carbohydrate responsive element binding protein in the control of ethanol metabolism and susceptibility to binge drinking. *Hepatology*.

[99] Zeng T., Zhang C.-L., Zhao N. (2018). Impairment of Akt activity by CYP2E1 mediated oxidative stress is involved in chronic ethanol-induced fatty liver. *Redox Biology*.

[100] Yu B., Qin S.-y., Hu B.-l., Qin Q.-y., Jiang H.-x., Luo W. (2019). Resveratrol improves CCL4-induced liver fibrosis in mouse by upregulating endogenous IL-10 to reprogramme macrophages phenotype from M(LPS) to M(IL-4). *Biomedicine & Pharmacotherapy*.

[101] Sun Z.-L., Zhang Y.-Z., Zhang F. (2018). Quality assessment of *Penthorum chinense* Pursh through multicomponent qualification and fingerprint, chemometric, and antihepatocarcinoma analyses. *Food & Function*.

[102] Ceni E., Mello T., Galli A. (2014). Pathogenesis of alcoholic liver disease: role of oxidative metabolism. *World Journal of Gastroenterology*.

[103] Qin L., Wang Z., Tao L., Wang Y. (2010). ER stress negatively regulates AKT/TSC/mTOR pathway to enhance autophagy. *Autophagy*.

[104] Zhang Y., Cao Y., Chen J., Qin H., Yang L. (2019). A new possible mechanism by which punicalagin protects against liver injury induced by type 2 diabetes mellitus: upregulation of autophagy via the akt/FoxO3a signaling pathway. *Journal of Agricultural and Food Chemistry*.

[105] Koga T., Suico M. A., Shimasaki S. (2015). Endoplasmic reticulum (ER) stress induces sirtuin 1 (SIRT1) expression via the PI3K-Akt-GSK3*β* signaling pathway and promotes hepatocellular injury. *Journal of Biological Chemistry*.

[106] Wang K. (2015). Autophagy and apoptosis in liver injury. *Cell Cycle*.

[107] Tomas-Hernandez S., Blanco J., Rojas C. (2018). Resveratrol potently counteracts quercetin starvation-induced autophagy and sensitizes HepG2 cancer cells to apoptosis. *Molecular Nutrition & Food Research*.

[108] Shaito A., Posadino A. M., Younes N. (2020). Potential adverse effects of resveratrol: a literature review. *International Journal of Molecular Sciences*.

[109] Wang Y., Tao B., Wan Y. (2020). Drug delivery based pharmacological enhancement and current insights of quercetin with therapeutic potential against oral diseases. *Biomedicine & Pharmacotherapy*.

